# A Method for the Evaluation of Early Osseointegration of Implant Materials Ex Vivo: Human Bone Organ Model

**DOI:** 10.3390/ma14113001

**Published:** 2021-06-01

**Authors:** Sergej Zankovic, Michael Seidenstuecker, Wolf C. Prall, Johannes Loos, Franziska Maderer, Mike Oberle, Sergio H. Latorre, Pia Schilling, Bianca Riedel, Anke Bernstein

**Affiliations:** 1 G.E.R.N. Tissue Replacement, Regeneration & Neogenesis, Department of Orthopedics and Trauma Surgery, Faculty of Medicine, Medical Center-Albert-Ludwigs-University of Freiburg, Hugstetter Straße 55, 79106 Freiburg, Germany; michael.seidenstuecker@uniklinik-freiburg.de (M.S.); sergio.latorre@uniklinik-freiburg.de (S.H.L.); pia.schilling@uniklinik-freiburg.de (P.S.); bianca.riedel@uniklinik-freiburg.de (B.R.); anke.bernstein@uniklinik-freiburg.de (A.B.); 2 Schoen Clinic Munich Harlaching, Teaching Hospital of Paracelsus Medical University Salzburg, 5026 Salzburg, Austria; christian.prall@yahoo.com; 3 Department of General, Trauma and Reconstructive Surgery, Munich University Hospital, Ludwig-Maximilians-University (LMU), Nussbaumstr. 20, 80336 Munich, Germany; 4 FIT Additive Manufacturing Group, FIT Production GmbH, Am Grohberg 1, 92331 Lupburg, Germany; Johannes.Loos@pro-fit.de (J.L.); Franziska.Maderer@pro-fit.de (F.M.); 5 RKK Klinikum, St. Josefskrankenhaus, Klinik f Unfallchirurgie, Orthopaedie, Kinder-und Sporttraumatologie, Sautierstr. 1, 79104 Freiburg, Germany; mike.oberle@rkk-klinikum.de

**Keywords:** organ model, 3D cell culture, bone, biomaterials, implant, osseointegration

## Abstract

In the present work, an ex vivo organ model using human bone (explant) was developed for the evaluation of the initial osseointegration behavior of implant materials. The model was tested with additive manufactured Ti6Al4V test substrates with different 3D geometries. Explants were obtained from patients who underwent total knee replacement surgery. The tibial plateaus were used within 24 h after surgery to harvest bone cylinders (BC) from the anterior side using hollow burrs. The BCs were brought into contact with the test substrate and inserted into an agarose mold, then covered with cell culture media and subjected to the external load of 500 g. Incubation was performed for 28 days. After 28d the test substrate was removed for further analysis. Cells grown out BC onto substrate were immunostained with DAPI and with an antibody against Collagen-I and alkaline phosphatase (ALP) for visualization and cell counting. We show that cells stayed alive for up to 28d in our organ model. The geometry of test substrates influences the number of cells grown onto substrate from BCs. The model presented here can be used for testing implant materials as an alternative for in vitro tests and animal models.

## 1. Introduction

Bone-cell adhesion to implant surfaces is the first step during early osseointegration. It affects the further bone formation and thus the success of the integration of implants into the bone [1]. The process of binding cells to implant surfaces is very complex. The properties of implant materials play a crucial role in regulating cell adhesion, proliferation, and differentiation [2]. Moderately hydrophilic surface (such as for example, Ti6Al4V alloy used for medical implants) found to be preferable for cells adhesion due to the adhesion-mediating proteins such as fibronectin and vitronectin [3,4,5,6]. Surface topography and roughness are important factors for cell adhesion and growth. In particular, nanorough surfaces have a positive effect on adhesion and growth osteoblasts [5,6,7,8]. A combination of surface free energy and surface topography and roughness influences cell-implant interaction, as was shown evaluating ZrO_2_ ceramics, titanium, Ti6Al4V, and CoCrMo alloy, which undergo different surface treatments such as polishing, sand-blasting, and etching [9]. Effect of surface chemistry and surface topography was also found in an animal model in vivo, where the coating of implant surface with natural biopolymers or plasma chemical treatment results in an enhanced implant anchorage and higher bone contact area in rats [10,11]. Up to now, there are two common methods for testing the cellular response toward biomaterials, in particular, in vitro cell culture and animal models. In vitro tests include but are not limited to tests of cell adhesion, cytotoxicity, metabolic activity, and proliferation. Cytotoxicity tests provide information on the ability of the substrate to damage cells or, in other words, evaluate the living cell’s reaction to the material. Proliferation assays, such as MTT metabolic assay, are used for analyzing proliferation, viability and cytotoxicity according to ISO 10993–5:2009 [12]. These methods have some important bottlenecks. In vitro testing has a disadvantage that cells are not organized in tissues and therefore are not interacting with each other and with surface in the same manner as in vivo. Animal model has a disadvantage that the animals are physiologically different from human. Recently, an organ model is considered to be a suitable method to overcome the disadvantages of cell cultures and animal models. It is possible to represent the bone as lifelike as possible in vitro.

The difficulty here is to maintain the function of the bone over a longer period of time, ensuring supplying bone cells with culturing medium. Moreover, mechanical load similar to the physiological environment must be established during culturing. Several studies have shown that it is possible to keep bone cells alive for a time in bioreactors [13,14,15]. Such bioreactors realize the perfusion of medium through bone tissue and provide a possibility to evaluate cell and tissue response to the different culturing conditions, e.g., to the mechanical load. For example, human femoral head core exhibit higher osteoblast function and higher viability of osteocytes up to 27 days under dynamic load [16]. Other bioreactors use hydrostatic pressure and did not provide medium perfusion through the tissue. The limitation of such organ models is the weak repopulation with the very few cells and, therefore, a need for additional seeding of osteoblasts, cultured in vitro [17]. The other limitation of reported ex vivo organ models is that bone tissue does not contain any vasculature and contain only the trabecular part of the bone [14,17,18]. Finally, up to now no studies reported an analysis of the outgrowth of bone cells onto implant surface using organ models.

The aim of this work was to develop an organ model using human bone to study the early adhesion of cells to implant surfaces. The model was evaluated using Ti6Al4V substrates with three different surface textures. Ti6Al4V alloy is a commonly used material for orthopedic implants because of its good biocompatibility, high corrosion resistivity, and high fatigue strength [19]. The test substrates were fabricated using 3D additive manufacturing technique and represent the surface of the tibial part of total knee implant. We hypothesized that it is possible to keep bone cells alive for 28 days and that cells outgrowth onto test substrate are affected by substrate texture. The use of such bone model for the analysis of local implant-tissue interaction would enable the analysis of the healing behavior of implants in the bone.

## 2. Materials and Methods

Agarose was obtained by SERVA (SERVA Electrophoresis GmbH, Heidelberg, Ger-many). Dulbeccos modified eagle medium (DMEM), fetal bovine serum (FBS), ethanol, Triton X-100, Penicillin/Streptomycin (P/S), Paraformaldehyde (PFA) and TUNEL assay kit were obtained from Sigma Aldrich (Sigma Aldrich (now Merck), Darmstadt, Germany). Phosphate buffered saline (PBS, GIBCO) was obtained from Fisher Scientific GmbH. Bovine serum albumin (BSA) was obtained from AppliChem (AppliChem GmbH, Darmstadt, Germany). The primary antibodies Collagen-I (col-I) (ab6308) and alkaline phosphatase (ALP) (ab65834) were obtained by Abcam (Abcam, Cambridge, UK). The secondary anti-bodies IgG Alexa Fluor 568 (A11011) and IgG Alexa Fluor 488 (A11001) were obtained by ThermoFisher (Thermo Fisher, Waltham, MA, USA). 4,6-Diamidino-2-phenylindole (DAPI) solution was obtained by Carl Roth (Carl Roth GmbH + Co. KG, Karlsruhe, Germany). The LowCross buffer was obtained by Candor (Candor bioscience GmbH, Wangen im Allgaeu, Germany). Technovit 9100 neu Kit was obtained from Kulzer (Kulzer GmbH, Hanau, Germany).

The Plexiglas rod (d = 15 mm) was obtained from Sigma Aldrich (Sigma Aldrich, Darmstadt, Germany). 50 mm petri dishes (deep) were obtained from Thermo Scientific (Thermo Fisher, Waltham, MA, USA).

### 2.1. Preparation of the Agarose Molds

**Figure 1:**
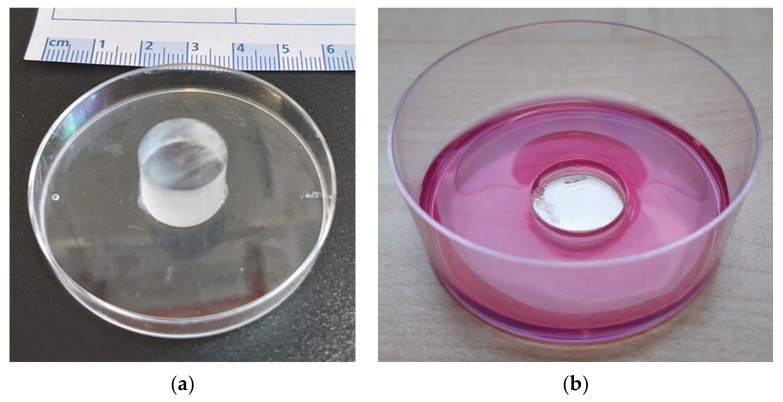
Plexiglas stamp for mold preparation (**a**) and the agarose mold (**b**). Bone cylinder will be placed into the hole in the middle.

For the organ culture model, a stamp was made in advance from a Plexiglas rod (d = 15 mm, l = 19 mm) and a top of a Petri dish by gluing. 4% wt agarose was prepared with DMEM and autoclaved. The Petri dish bottoms were sterilized with 70% ethanol and al-lowed to evaporate under the sterile bench. The still hot agarose/DMEM was then mixed with DMEM in which 20% FBS and 2% P/S were dissolved. This gives a final DMEM + 10% FBS + 1% P/S + 2% agarose solution, of which 7 mL each was poured into the Petri dish bottoms while hot. Immediately after, the stamp (see Figure 1) was put on and everything was allowed to cool well. The molds thus obtained were used for culturing the organ cultures.

### 2.2. Fabrication of Titanium Disks

**Figure 2:**
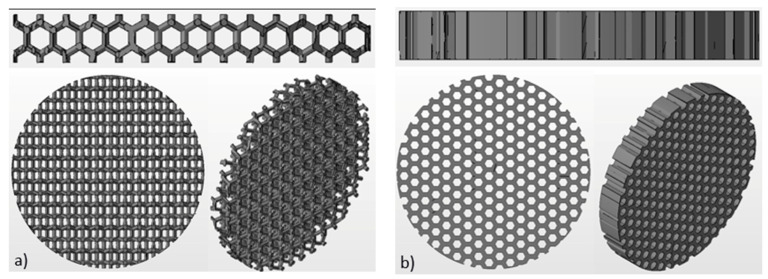
Schematic images of 3D structure used for the fabrication of Ti-disks: (**a**) wurtzite and (**b**) honeycomb. Disks have the diameter of 15 mm and are 2 mm thick.

Ti6Al4V disks (is hereinafter referred to as Ti-disks) were fabricated using 3D additive manufacturing technique, namely electron beam melting (EBM). An Arcam Q10 (Mölnlycke, Sweden) system was used for this purpose. The Arcam Q10 is the 3rd generation of EBM machines and was especially developed for the cost-efficient production of standard and customized implants. It consists of two main components: the control cabinet with operator interface and the vacuum chamber with the electron beam source and the powder dosing system as well as the build platform. The maximum build size is 200 × 200 × 180 mm (W × D × H). Titanium disks with a diameter of 15 mm and thickness of 2 mm were fabricated for this study. Three different textures were fabricated: solid (non-structured as control), honeycomb and wurtzite structure (see Figure 2).

3D data were delivered by a CAD system and processed on a STL-based model. If necessary, support structures were attached. The support is primarily used for heat dissipation during the build-up process, but also to support and anchor the component on the build platform. The models with support structures were then arranged in the build space according to specific criteria to ensure optimum heat distribution. In this process, it is possible to place the components on top of each other (“stacking”). In the last step of data preparation the models were sliced into several 2D layers.

In the subsequent building process the Ti-disks are melted layer by layer from the metal powder by an electron beam, whereby the model is created gradually from the individual slices. The EBM process takes place in a vacuum at elevated temperatures (~650 °C). In the build chamber are two powder containers which contain the metal powder. A rake spreads a thin layer of powder (50 µm) on the build platform. The electron beam is directed onto this platform and previously defined areas are locally melted. After this step, the build platform is lowered, a new powder layer is applied and the build process is repeated layer by layer until the complete part has been produced. The build speed here is 3–4 mm/h. The vacuum prevents the entrapment of oxygen. After cooldown the build platform was removed. The finished Ti-disks were blasted out of the sintered powder block using Ti6Al4V powder, so that the powder can be reused after sieving. The support structures were removed and the Ti-disks were polished if necessary.

Powder of Ti6Al4V Grade 5 with grain size from 45 µm to 106 µm was used as material for the disks. Due to the distribution of the different grain sizes the powder has an apparent density of 2.47 g/cm^3^. The main chemical element of Ti6Al4V alloy is titanium together with 6% aluminum and 4% vanadium [20]. Chemical composition of Ti6Al4V alloy according to the manufacturer’s material data sheet is summarized in Table 1. The material has a yield strength (Rp0.2) of 950 MPa, a tensile strength (Rm) of 1020 MPa, the modulus of elasticity is 120 GPa, according to the manufacturer’s material data sheet [21]. The mechanical properties of components produced in the EBM process are comparable to wrought annealed materials and are more resilient than cast materials.

### 2.3. Sample Aquisition

**Figure 3:**
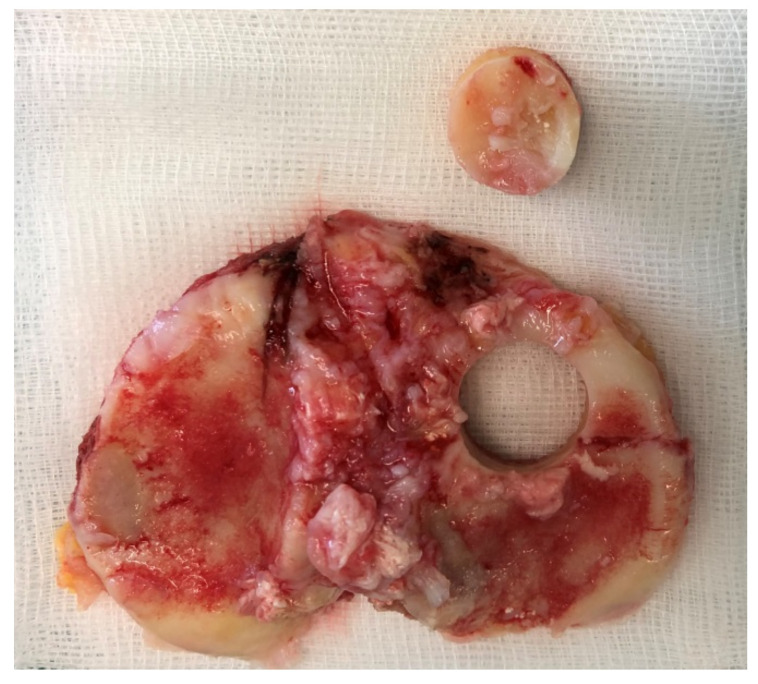
Bone cylinder harvested from tibia plateau.

**Figure 4:**
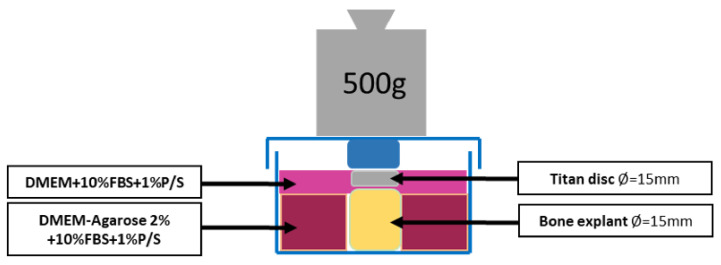
Schematic diagram of the organ model.

Tibial plateaus from patients who underwent TKR and corresponded to osteoarthritis grade 4 according to Kellgren and Lawrence [22] were used for organ cultures. A positive ethical vote of the ethics committee of the Albert Ludwig University of Freiburg was granted (420/19). The tibial plateaus were used within 24 h after surgery to harvest bone cylinders (BC) from the anterior side (with the cartilage) using hollow burrs. To ensure that the patient’s bone was still alive, we allowed osteoblasts to grow out in well-plate at time zero. If no cells grew after 10 days, the sample was discarded. Harvesting of the BCs was performed under sterile conditions. After harvesting, any cartilage tissue was removed from the BCs and the BCs were placed in a stainless steel mold with a central hole of d = 15 mm into which the bone cylinders were inserted. The hole has a depth of 5 mm so that all BCs can be sawed to an equal height of 5 mm (Figure 3). The obtained BCs are washed three times with PBS + 1% P/S to remove the fat. Then one BC at a time was placed in the previously prepared mold. 3D powder-printed titanium disk (d = 15 mm) with various mesh structures was placed onto the BC. DMEM + 10% FBS + 1% P/S was applied until everything is covered. Incubation in the incubator was performed with the stamp (that was used to produce the molds) at 500 g weight per each BC for 28d (see Figure 4). The medium was changed every 7d. In this process, 3 mL of medium was removed and replaced with 4 mL of fresh medium.

A total of 11 tibia plateaus were collected for this study. Samples came from male and female patients with an age range between 60 and 85. Twenty-one bone cylinders were prepared. These cylinders were randomly divided into three groups for culturing with solid-, wurtzite- and honeycomb-disks, i.e., seven BC per group.

### 2.4. Immunostaining

Then, 28d titanium disks were removed from the culturing medium and processed. The disks were washed three times in PBS, fixed using 4% PFA for 15 min and washed again three times in PBS. Blocking was performed with 1% BSA solution in PBS for 45 min, followed by washing in PBS three times. The primary antibodies were diluted in LowCross buffer 1:100 and the secondary antibodies in LowCross buffer 1:500. Primary antibodies (Col-I, ALP) were applied to the disks overnight at 4 °C. After washing three times in PBS, secondary antibodies were applied for 60 min at room temperature. DAPI solution (1 µg/mL) was applied for 10 min, followed by washing three times in PBS and mounted in DABCO. Disks were stored in 0.04% AZIDE before analyzing.

Cryosections of bone cylinders were dried at air for 30 min, fixed with 4% PFA for 30 min and washed three times in PBS. Then, BCs were permeabilized in 0.1% Triton/PBS solution for 30 min. TUNEL solution was prepared from 50 µL of enzyme solution and 450 µL label solution. BCs were incubated in TUNEL solution at 37 °C for 1h and subsequently washed three times in PBS. Finally, BCs were mounted with DABCO-DAPI.

### 2.5. Microscopy

Immunostained cells on titanium disks were imaged using Olympus BX51 fluorescent microscope equipped with 10× and 20× objectives (Olympus, Tokyo, Japan). Images with the resolution of 2560 × 1920 pixels were recorded at 8 different positions on each disk. Cell counting was performed using ImageJ 1.47 software.

### 2.6. Statistical Analysis

Data (number of cells) are presented as box-and-whisker plots indicating median, quartiles, whiskers, and outliers. One-way analysis of variance (ANOVA) was performed for all three groups followed by multiple comparisons with Fisher’s LSD test with the significance level of 0.05 Statistical analysis was performed using Origin Pro 2021 9.8 Software (OriginLab Corporation, Northampton, MA, USA).

## 3. Results and Discussion

**Figure 5:**
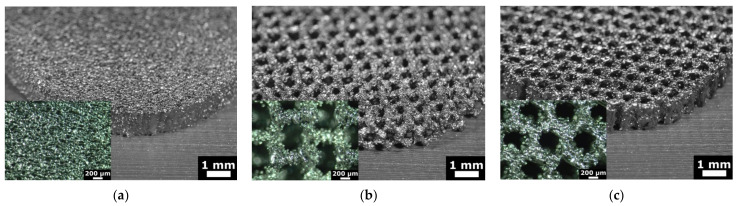
Microscopy images of Ti-disks fabricated using 3D additive manufacturing technique: (**a**) solid, (**b**) wurtzite and (**c**) honeycomb. Disks have the diameter of 15 mm and are 2 mm thick.

The EBM process enables the production of complex geometries without the limitations of conventional manufacturing processes. Unique, fine surface structures that support osseointegration can be produced. Such structures are, for example, 3D porous mesh structures. Pore sizes of 200–600 μm are generally considered favurable for bone ingrowth as reported previously [23,24,25]. Therefore, we have fabricated test substrates with three different structures to validate bone organ model. These were solid (non-structured), wurtzite and honeycomb. Images of the test substrates fabricated using additive manufacturing technique are shown in Figure 5. Solid substrate represents a commonly used surface of orthopedic implants and serves as a control. Two others are porous textured substrates. The design and geometrical values were determined by CAD system. The geometry of the additive manufactured disks was inspected using optical microscopy. The size and the shape of rods had deviations up to 100 µm, while the pitch and symmetry was reproduced well (as can be seen in Figure 5). This result was expected, since powder with grain size from 45 µm to 106 µm was used for manufacturing (see Section 2.2 Fabrication of titanium disks). The average size of the mesh openings visible at the top projection view was around 400 µm at the narrow place, as measured using microscopy images. Wurtzite is a 3D open pore structure, while honeycomb is open only to the top and bottom surface of the disk. It should result in an enhanced diffusion of the culturing medium to the bone-implant interface. Therefore, these mesh structures were chosen to test and validate our bone organ model. Mechanical properties of these mesh structures were not accessed in this study and were not considered by choosing mesh design.

The potential of implant biomaterials for the integration with bone can be usually evaluated using examination of its biocompatibility, i.e., testing the potential of the biomaterial to support cell adhesion, viability, and proliferation. Using an organ model has a potential to evaluate biomaterials at lifelike environment, such as mechanical load or dynamic supply of cells with nutrition.

**Figure 6:**
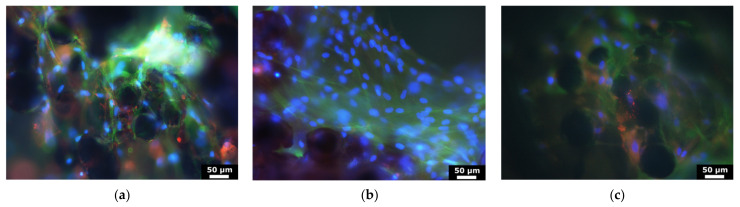
Microscopy images of cells, which are grown out of BC onto Ti-disk with different surface textures: (**a**) solid Ti-disk, (**b**) wurtzite and (**c**) honeycomb. Cells were immunostained with alkaline phosphotase (red), collagen-I (green) and DAPI (blue) prior imaging.

**Figure 7:**
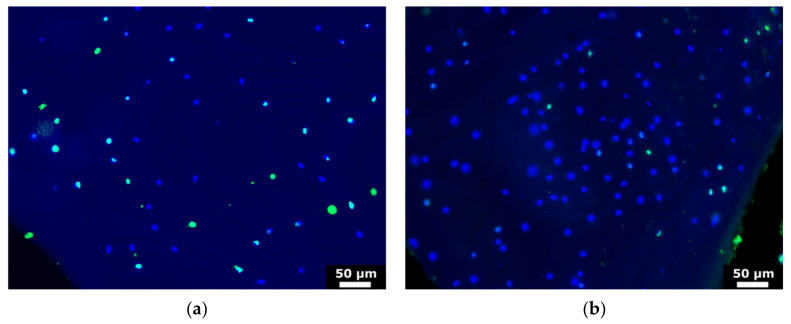
Microscopic images of the (**a**) longitudinal and (**b**) transversal view of bone cylinder stained with TUNEL assay. The viable osteocytes are labeled blue, dead osteocytes are labeled green.

To validate our bone organ model, cells which were grown out of bone cylinders onto Ti-disks were analyzed after 28 days. Cell cores were stained with DAPI, cells were immunostained for type I collagen and ALP (osteoblast markers) and imaged. Typical images recorded with the magnification of 20× are shown in Figure 6. These results suggested that cells are viable after 28d culturing and express both collagen-I and alkaline phosphatase. The viability of the cells within the bone was confirmed using TUNEL test of bone cylinders before and after the culturing. The results suggested that osteocytes were staying alive after 28d culturing, as can be seen in Figure 7, where viable osteocytes are labeled blue and dead osteocytes are labeled both blue and green.

**Figure 8:**
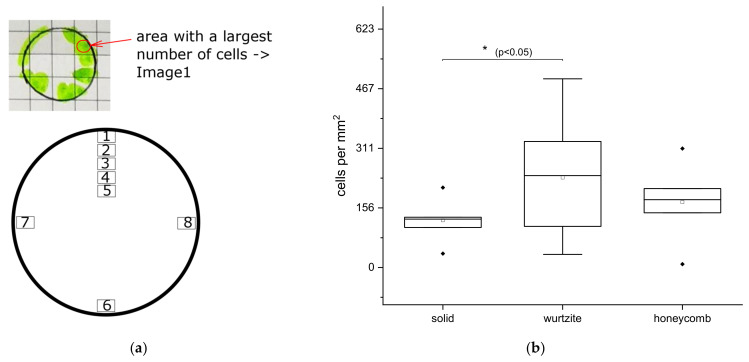
Schematics of 8 analyzed areas on Ti-disks: green areas represent cells on Ti-disks (**a**). Cells were counted inside areas 1–8 (**a**). Number of the cells grown out from bone cylinder onto Ti-disks with different surface textures (**b**). Number of cells was calculated using seven bone cylinders per group and one repetition.

To quantify the early cell adhesion, the number of cells was counted as follows. An area with the largest number of cells was marked as area 1 (Figure 8a). Starting from this area, four further areas with offset of 1 mm toward the center of disk (areas 2–5 in Figure 8a) were then selected. In addition, three areas were selected from the three opposite sides of disk (6–8 in Figure 8a). Images were taken from all these areas at the same microscopic resolutions and at the magnification of 10× and the cell number within these areas was counted. Results corrected for area are shown in Figure 8b. The largest number of cell outgrowth from the bone onto test substrate was found on Ti-disk with wurtzite structure (statistically significant different from solid Ti-disk). About 39% more cells were grown on the disk with honeycomb structure compared to solid disk, but there is no statistically significant difference.

We assume that this can be attributed to both overall surface size of the disks and with the supply the cells with culturing medium. Since wurtzite structure is an open porous structure, the culturing medium can easily perfuse and supply the bone-implant interface with the nutrition. Honeycomb structure is open only to the top and to the bottom side of the disk that leads to the disturbed diffusion of culturing medium. Moreover, the diffusion is additionally disturbed due to the weight on the top. Cells can be supplied with nutrition only due to diffusion through the cancellous bone cylinder. Therefore, cells tended to grow only on the surface of honeycomb structure.

Other studies with different ex vivo models have also reported the loss of cell viability due to worse diffusion of culturing medium. Mechanical loading, static or cyclic, is important to maintain bone response [16,17]. Increased cell viability in bioreactor using medium perfusion through vasculature of femoral head was reported [18]. Some other models attempt to mimic bone remodeling in vitro [26], evaluate the viability of bone cells within 3D scaffolds [27,28,29] or used humanized mice bone model [30,31]. Nevertheless, these studies have not considered the bone cells on the bone-material interface.

Our results demonstrated that human bone organ model described here allow evaluation of the effect of implant material on the cell adhesion and, thus, early osseointegration ex vivo in a lifelike environment. Finally, these results suggested that wurtzite structure, applied to the back side of the implant, could affect the early osseointegration positively, compared to the non-structured solid surface.

Previously described research used organ models to study osteoblasts and osteocytes activities under hydrostatic pressure [17], apoptosis and osteocytes activity under cyclic load [16], ex vivo bone remodeling [13] or trabecular bone stiffness [32] using human, sheep and bovine explants. The innovation of present work is the evaluation of the bone cells outgrowth from bone explant onto an implant material, which was brought into contact with bone cylinder. We used human bone from patients receiving endoprostheses for osteoarthritis. During implantation of prosthesis, the implant would come into contact with the bone and this situation is exactly reproduced in our organ model. Thus, it is possible to evaluate the implant performance from the point of view of osseointegration. The limitation of the model presented here is that it does not mimic an optimal physiological condition. In particular, we do not realize physiological diffusion of the medium. Only static mechanical load was realized in this work. Further optimization of organ model would be of interest to avoid limitations mentioned here. Moreover, RNA expression analysis (for example, runx2 and ALPL) could be also performed in the future. Nevertheless, our model could be used for the testing of different implant materials for their osseointegration performance. In addition, these bone organ cultures maintained the natural position of osteocytes within the extracellular mineralized matrix. Moreover, ex vivo bone organ cultures might allow also the study of infection as well as wear debris on bone cells.

## 4. Conclusions

We established an ex vivo organ model using human bone (explant) for the evaluation of the initial osseointegration behavior of implant materials. Cells stayed alive for up to 28 days in our organ model. The number of cells grown onto the substrate from bone cylinders depended of the 3D geometry of test substrates. The model presented here could serve as an alternative for in vitro tests and animal models for testing implant materials.

## Figures and Tables

**Table 1:** Chemical composition of Ti6Al4V used for fabrication of Ti-disks.

Al	V	C	Fe	O	N	H	Ti
5.5–6.5%	3.5–4.5%	<0.08%	<0.25%	<0.13%	<0.05%	<0.012%	Balance

## Data Availability

The data presented in this study are available on request from the corresponding author.
